# COVID-19 Pathogen Viral Evolution Leading to Increased Infectivity

**DOI:** 10.7759/cureus.26660

**Published:** 2022-07-08

**Authors:** Sonam Parag, Katelyn Carnevale

**Affiliations:** 1 Dr. Kiran C. Patel College of Allopathic Medicine, Nova Southeastern University, Fort Lauderdale, USA

**Keywords:** sars-cov-2 (severe acute respiratory syndrome coronavirus-2), middle east respiratory syndrome (mers-cov), corona virus disease 2019, corona virus, covid-19, spike protein, sars-cov

## Abstract

Objective

This study investigated changes in viral protein structures within the receptor-binding domains (RBDs) of the viral particles of severe acute respiratory syndrome coronavirus (SARS-CoV), Middle East respiratory syndrome coronavirus (MERS-CoV), and severe acute respiratory syndrome coronavirus 2 (SARS-CoV-2), that may explain the evolution of increased infectivity.

Background

The emergence of severely pathogenic *Betacoronaviruses* indicates increased infectivity and host range, possibly related to the evolution of the viral genome and subsequent proteins, specifically coronavirus spike proteins that are involved in host receptor binding and cell entry.

Methods

Amino acid sequences of the spike protein of each virus (SARS-CoV, MERS-CoV, and SARS-CoV-2) were obtained from the NCBI Virus Database, along with the sequences for their known receptors, and analyzed for sequence changes and peptide properties to determine the characteristics of the virus-receptor binding. Crystal structures were retrieved from the Protein Database for each virus and receptor and visualized using proteomic analysis software (PyMOL 2.1) (Schrödinger, Inc., New York, USA).

Results

SARS-CoV-2 displayed the largest magnitude difference (+32.4) in net charge between the virus and its receptor, angiotensin-converting enzyme 2 (ACE2), suggesting stronger electrostatic binding. SARS-CoV-2 also had the largest RBD (7140.29 Å^2^), indicating more surface area for interaction with the ACE2 receptor.

Conclusion

The evolution of SARS-CoV-2 for a larger and more electrostatically “sticky” RBD compared to other pathogenic *Betacoronaviruses* may contribute to observations of SARS-CoV-2 having a stronger or more stable binding, leading to increased transmissibility and infectivity. Further investigation of conserved genomic regions between these viruses may facilitate the development of viable vaccines and treatments.

## Introduction

Human coronaviruses, known for their iconic surface spike proteins, were first identified in the mid-1960s by D. A. J. Tyrrell, who was leading a group of virologists working with human and animal viral strains [1]. This group of viruses was not considered to be highly pathogenic until the outbreak of severe acute respiratory syndrome (SARS) caused by the *Betacoronavirus* severe acute respiratory syndrome coronavirus (SARS-CoV) in 2003 [2]. The SARS virus, assumed to have an animal host origin, was first isolated in Guangdong, China, and quickly spread around the world [3]. This virus rapidly disappeared, despite having a replication rate (R_0_) of between two and four, according to the World Health Organization [4]. Nine years after the emergence and disappearance of SARS-CoV, a related virus in the genus of *Betacoronavirus*, the Middle East respiratory syndrome coronavirus (MERS-CoV), emerged in 2012, assumed to originate from camels [3]. While Middle East Respiratory Syndrome (MERS) was a more lethal disease than SARS, MERS-CoV was a less transmissible virus than SARS-CoV, making it self-limiting with a R_0 _of less than one [5]. A key difference between these two viruses is that the SARS-CoV spike protein uses angiotensin-converting enzyme 2 (ACE2) as a receptor for cellular entry, a multifunctional protein specifically known for its role in the renin-angiotensin-aldosterone system. While MERS-CoV spike protein uses dipeptidyl peptidase 4 (DPP4) as a receptor for viral entry [3]. This receptor is multifunctional but largely known for its role in human glucose metabolism [6].

In the winter of 2019, the *Betacoronavirus* genus made another leap from animals to humans, this time in Wuhan, China, leading to the emergence of Coronavirus Disease 2019 (COVID-19), caused by the Severe Acute Respiratory Syndrome Coronavirus 2 (SARS-CoV-2), leading to the worst pandemic our world has seen in recent memory, with a current global death toll of 6,320,697 (as of June 21, 2022) [7,8]. SARS-CoV-2 has been shown to also interact with the ACE2 receptor for viral attachment, however, this viral species seems much more contagious than its predecessors, with a R_0_ ranging from three to six [9]. The concerted efforts of scientists around the globe have led to a large collection of viral sequences on global databases, which have allowed for the direct observation of SARS-CoV-2 viral evolution as it is rapidly mutating during continuous transmission among humans.

Coronavirus is comprised of four structural proteins: spike, envelope, membrane, and nucleocapsid proteins [3]. Among these, the spike protein is of note because it plays an important role in the initiation of the infectious process and serves as a target for the development of antibodies and vaccines. The coronavirus spike protein is a trimeric glycoprotein on the viral envelope that binds to cellular receptors, triggering fusion and cell entry [3]. Significant mutations have been identified in the spike protein of SARS-CoV-2 and could possibly be the source for the observation of high infectivity. Scientists are still unsure of the biological determinants of mortality versus infectivity and transmissibility of these related viral species. Tracking changes in the receptor-binding domain (RBD) of the spike protein within the various *Betacoronavirus* strains may help provide a better structural understanding of each virus to guide the development of viable vaccines and therapeutics to combat the current pandemic as well as future pathogenic mutations.

## Materials and methods

This study investigated the viral genomic changes leading to changes in protein structure in the RBD of the viruses responsible for SARS, MERS, and COVID-19, using available sequences from the NCBI Virus Database. The protein sequences were then evaluated for changes in amino acid composition and properties, as well as RBD size for SARS-CoV, MERS-CoV, and SARS-CoV-2, and their known receptors, to determine how binding might affect cellular entry and infectivity between these viral species.

Spike protein reference sequences

Full-length amino acid reference sequences of the spike protein for each virus were retrieved from the NCBI Virus Database, a dynamic community portal for viral sequence data from RefSeq, GenBank, and other NCBI repositories. Sequences acquired for RBD multiple sequence alignment analysis included spike glycoprotein SARS coronavirus (accession YP_009825051), spike protein Middle East respiratory syndrome-related coronavirus (accession YP_009047204), and surface glycoprotein severe acute respiratory syndrome coronavirus 2 (accession YP_009724390). These sequences were chosen after refining search results for the following characteristics: RefSeq sequence type, complete genome sequences, spike protein, and homo sapiens host. Comparisons were made to the amino acid sequence identity between the RBDs of the three viral species' spike proteins, and the chemical properties of each RBD were calculated using the Innovagen peptide property calculator (Innovagen AB, Lund, Sweden) [10].

Spike protein receptor-binding domain mutational analysis

Identification of mutations within the RBD of the spike protein for each virus was accomplished by multiple alignments of the three reference sequences (accession numbers: YP_009825051, YP_009047204, YP_009724390) using the NCBI basic local alignment search tool (BLAST) program under default settings. The graphic summary results from the BLASTP jobs demonstrated the detailed identification of conserved domains within each sequence. Each virus’s specific receptor-binding domain interval was identified from this search and compared for amino acid differences using the NCBI Multiple Sequence Alignment Viewer 1.22.0, where segments of non-homogeneity were manually counted and summed to determine the net sequence divergence between each of the three viral species.

Spike protein receptor-binding domain structural analysis

*Betacoronavirus* spike protein crystal structures were retrieved from the Protein Data Bank (PD IDs 5XLR, 5X5C, and 7DK3 for SARS-CoV, MERS-CoV, and SARS-CoV-2, respectively). They were then analyzed using PyMOL (Schrödinger, Inc., New York, USA) for molecular visualization and animation of three-dimensional structures and measurement of the physical parameters of the RBD for each spike protein. These methods were used to compare macroscopic structural changes between residues of interest within the receptor-binding domain of each virus. Among these, the size dimensions of each binding domain were measured, as well as the three-dimensional orientation of the amino acids present in the binding site.

Conserved sequence analysis of SARS-CoV-2

Using the NCBI Virus database, the first 100 spike protein sequences of SARS-CoV-2 were selected and aligned using the multiple sequence alignment feature on the NCBI Virus webpage. These sequences were aligned to the reference sequence YP_009724390 to highlight differences between each sequence.

## Results

Spike protein sequences

**Figure 1:**
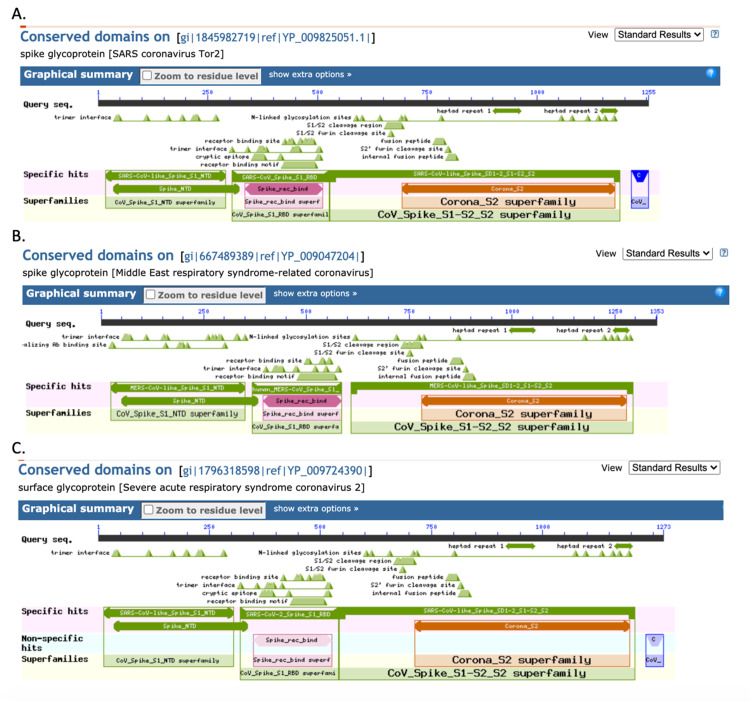
Spike protein receptor-binding domain identification: (A) SARS-CoV, (B) MERS-CoV, and (C) SARS-CoV-2. SARS-CoV: severe acute respiratory syndrome coronavirus, MERS-CoV: Middle East respiratory syndrome coronavirus, and SARS-CoV-2: severe acute respiratory syndrome coronavirus 2.

To track the evolution of amino acid changes among the selected members of the *Betacoronavirus* genus, the spike protein sequence was extracted and compared among SARS-CoV, MERS-CoV, and SARS-CoV-2. The RBD was the specific area of interest for tracking evolutionary mutations and analyzing sequence changes and homology. The RBD sequence within each virus’s spike protein was identified via a BLAST search and gave the following results: SARS-CoV RBD residues 306-527, MERS-CoV RBC residues 368-586, and SARS-CoV-2 RBD residues 319-541 (Figure 1A-1C).

**Figure 2:**
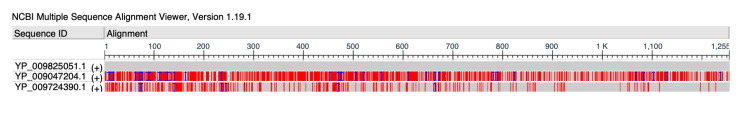
Multiple sequence alignment of SARS-CoV, MERS-CoV, and SARS-CoV-2. SARS-CoV: [YP_009825051.1], MERS-CoV: [YP_009047204.1], and SARS-CoV-2: [YP_009724390.1]. SARS-CoV: severe acute respiratory syndrome coronavirus, MERS-CoV: Middle East respiratory syndrome coronavirus, and SARS-CoV-2: severe acute respiratory syndrome coronavirus 2.

SARS-CoV was used as the “master sequence” for comparison, considering it appeared first evolutionarily. SARS-CoV was set as the master sequence to align the other two viruses. The coloring for the alignment was set to “show differences.” This method highlights differences observed from the master sequence in an alignment. Therefore, the red lines demonstrate differences in each virus compared to SARS-CoV (Figure 2).

A multiple sequence alignment of the spike protein of each virus demonstrated that MERS-CoV showed 30.63% homology to SARS-CoV with 785 mismatch base pairs, and SARS-CoV-2 displayed 76.19% homology to SARS-CoV with 278 mismatch base pairs (Table 1). By incorporating these values into the multiple sequence alignment, we can quantify the visual results of the highlighted differences seen in Figure 2.

**Table 1:** Viral sequence percent identity to the master sequence, percent coverage to the master sequence, and the number of mismatches to the master sequence. SARS-CoV: severe acute respiratory syndrome coronavirus, MERS-CoV: Middle East respiratory syndrome coronavirus, and SARS-CoV-2: severe acute respiratory syndrome coronavirus 2.

Organism	Identity (%)	Coverage (%)	Mismatches
SARS-CoV	100.00	100.00	0
MERS-CoV	30.63	96.57	785
SARS-CoV-2	76.19	99.68	278

**Figure 3:**
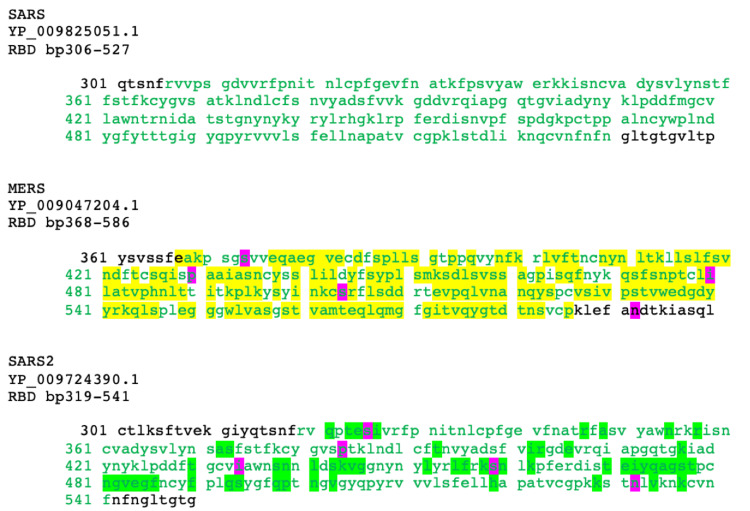
Spike protein RBD sequence alignment and manual identification of amino acid differences. The green font indicates residues included in the receptor-binding domain. The yellow highlights demonstrate the amino acid changes identified between SARS-CoV and MERS-CoV. Green highlights demonstrate the amino acid changes identified between SARS-CoV and SARS-CoV-2. Pink highlights demonstrate the same amino acid change at the same location from SARS-CoV to MERS-CoV and SARS-CoV to SARS-CoV-2. SARS-CoV: severe acute respiratory syndrome coronavirus, MERS-CoV: Middle East respiratory syndrome coronavirus, SARS-CoV-2: severe acute respiratory syndrome coronavirus 2, and RBD: receptor-binding domain.

A closer look at the RBD for each virus displayed that MERS-CoV exhibited 177 base-pair mutations compared to its predecessor, SARS-CoV, which emerged nine years earlier. SARS-CoV-2 exhibited 60 base-pair mutations from its predecessor SARS-CoV, with an emergence gap of sixteen years (Figure 3).

Spike protein receptor-binding domain chemical and physical property analyses

**Figure 4:**
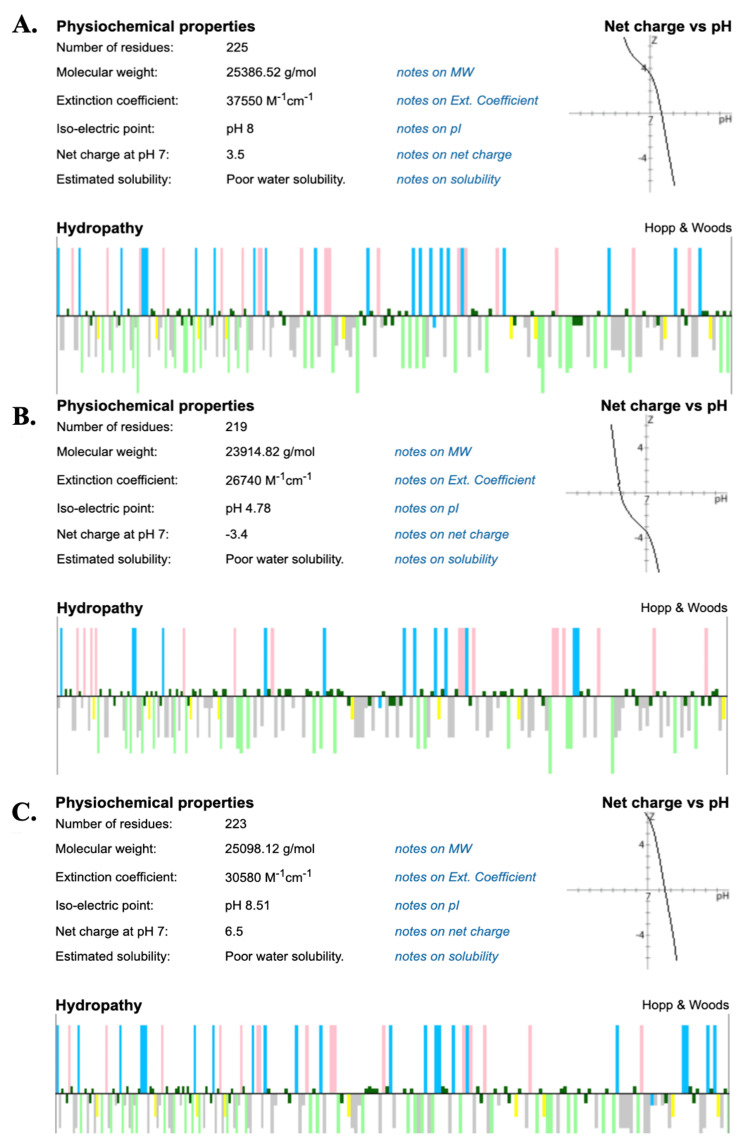
Peptide analyses of (A) SARS-CoV, (B) MERS-CoV, and (C) SARS-CoV-2. Hydropathy bar graph legend: Top is hydrophilic, bottom is hydrophobic, pink is acidic, neon green is aromatic, blue is basic, gray is aliphatic, dark green is polar, and yellow is cysteine. SARS-CoV: severe acute respiratory syndrome coronavirus, MERS-CoV: Middle East respiratory syndrome coronavirus, and SARS-CoV-2: severe acute respiratory syndrome coronavirus 2.

Comparing the spike protein RBD of each virus, SARS-CoV was found to have an isoelectric point at a pH of 8.00 and a RBD with a molecular weight of 25.387 kDa. MERS-CoV displayed an isoelectric point at a pH of 4.78 and a RBD with a molecular weight of 23.915 kDa. SARS-CoV-2 displayed an isoelectric point at a pH of 8.51 and a RBD with a molecular weight of 25.098 kDa (Figure 4).

**Figure 5:**
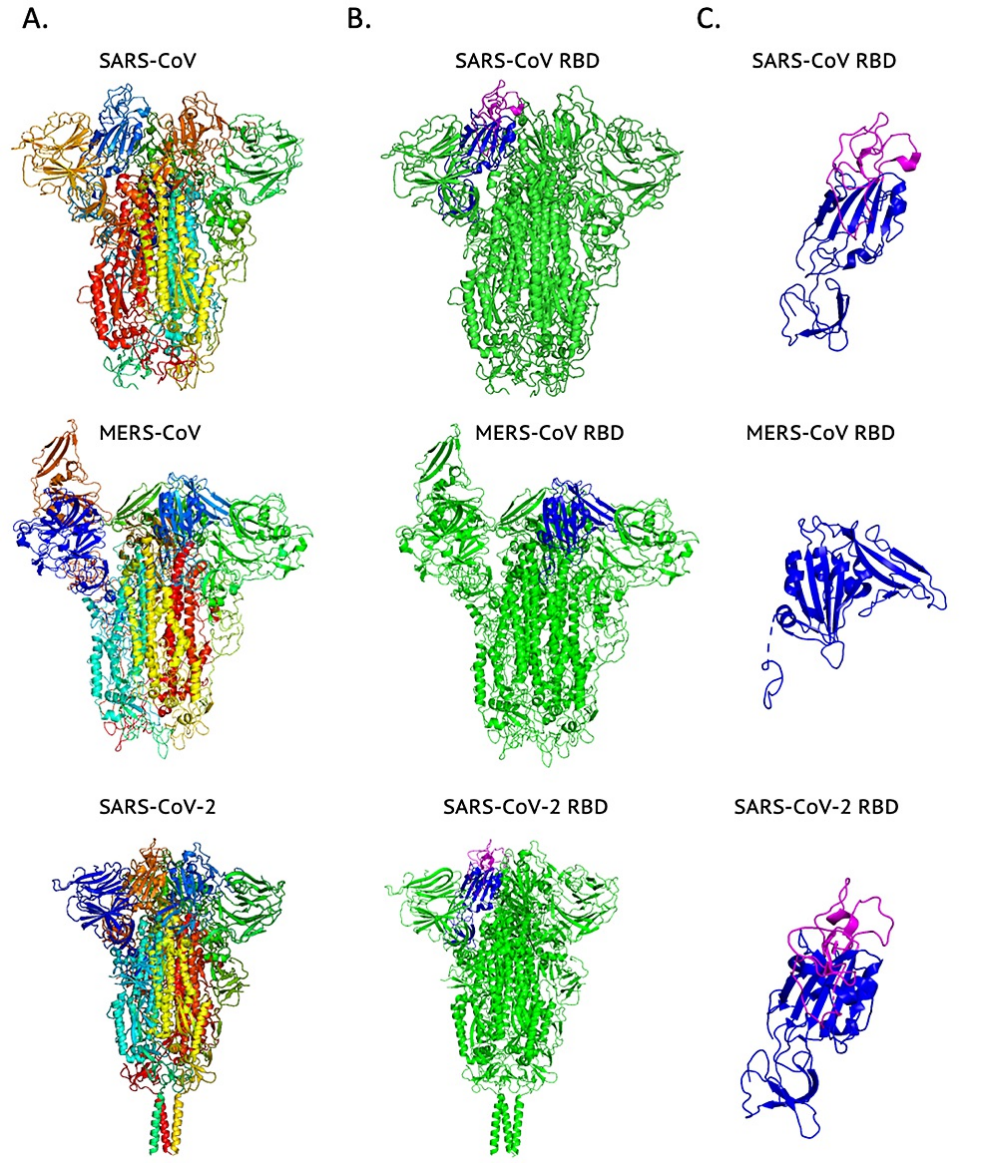
Three-dimensional representations of spike protein crystal structures. Column A shows the various secondary structure domains that constitute each virus's spike protein, with each conserved domain colored differently. Column B highlights the spike protein receptor-binding domain of each virus in blue and pink, noting that pink indicates the section that binds to the ACE2 receptor. Column C demonstrates the extracted receptor-binding domain for better visualization. SARS-CoV: severe acute respiratory syndrome coronavirus, MERS-CoV: Middle East respiratory syndrome coronavirus, SARS-CoV-2: severe acute respiratory syndrome coronavirus 2, and ACE2: angiotensin-converting enzyme 2.

Analysis of the protein crystal structures acquired from the Protein Database revealed that, on the macro-molecular level, all three structures demonstrated very similar structural homology for each spike protein (Figure 5).

SARS-CoV exhibited a RBD made up of eight beta sheets and three alpha-helices with a net +4 charge. The MERS-CoV RBD consisted of twelve beta sheets and six alpha-helices with a net charge of -2. SARS-CoV-2 displayed a RBD consisting of ten beta sheets and four alpha-helices with a net charge of +7. Finally, rudimentary surface area measurements were collected using the molecular ruler tool available in PyMOL to identify the relative sizes of each viral RBD. It was found that SARS-CoV-2 displayed the largest RBD surface area at 7140.29 Å^2^, compared to SARS-CoV (6685.11 Å^2^) and MERS-CoV (3455.17 Å^2^).

Conserved sequence analyses

**Figure 6:**
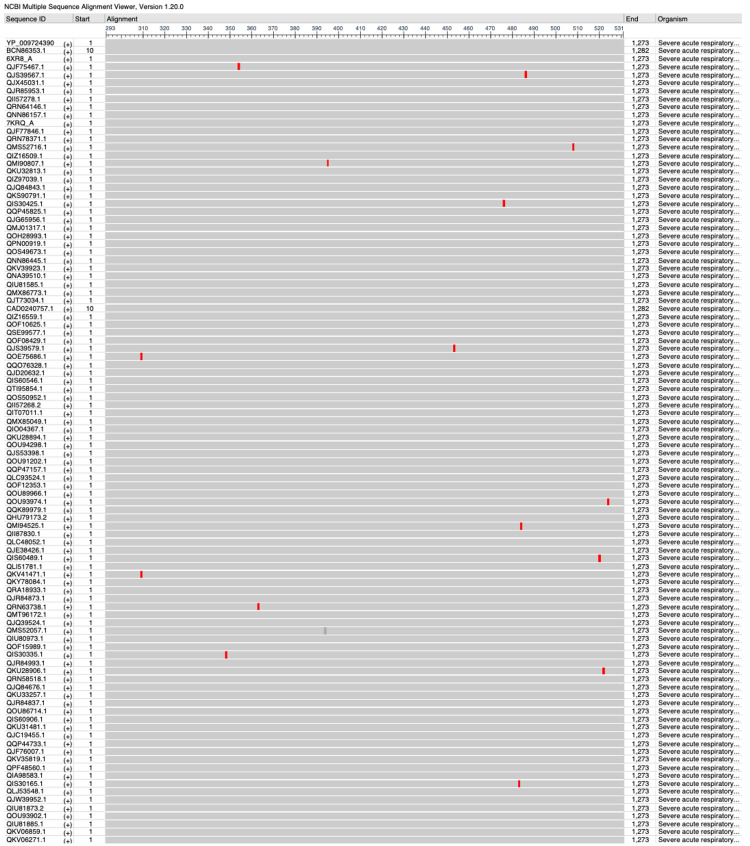
Identification of conserved regions of SARS-CoV-2. SARS-CoV-2: severe acute respiratory syndrome coronavirus 2.

After conducting an analysis of 100 spike protein sequences of SARS-CoV-2, several highly conserved regions were identified within the RBD that could be sites of interest for future studies as vaccine targets. Residues 319-347, 364-393, 396-452, 454-475, 486-507, and 525-541 could be possible areas of interest that have remained stable over time. Of note, residue 396-452 is the longest conserved region at 56 amino acids in length (Figure 6).

## Discussion

The recent outbreak of the SARS-CoV-2 infection has caused worldwide panic, sparking a large surge in research of the epidemiology, biochemistry, and immunology of this novel pathogen [3,11,12]. Understanding the biochemical nature of this virus can help scientists and clinicians to become better informed and equipped to successfully treat and prevent this deadly infection. To understand the genetic makeup of SARS-CoV-2, it is important to consider the viral origin and how it has evolved over time [1,2]. This project analyzed the predecessors of SARS-CoV-2, SARS-CoV, and MERS-CoV [2]. Although these different *Betacoronavirus* species evolved over a considerable amount of time and in different geographic locations, it seems that the virus responsible for the current COVID-19 pandemic shows significant sequence homology to its predecessor, SARS-CoV [13]. The multiple sequence alignment and RBD residue comparison demonstrated that SARS-CoV-2 and SARS-CoV differed only slightly, with 76.19% spike protein sequence homology. These results show that SARS-CoV-2 has a higher molecular similarity with SARS-CoV than MERS-CoV and further analysis of each RBD amino acid's chemical and physical properties may provide insight into the advantageous mutations that have allowed for SARS-CoV-2 to surpass SARS-CoV and MERS-CoV in infectivity and transmissibility leading to the current pandemic [4,5]. Based on the peptide analyses conducted, it can be inferred that the amino acid mutations that occurred between SARS-CoV and SARS-CoV-2 incorporated more basic, positively charged amino acids that may allow for better binding with the oppositely charged receptor ACE2, which has a highly negative charge [14]. The larger relative size observed using surface area measurements of the SARS-CoV-2 spike protein RBD compared to SARS-CoV may suggest a larger area for interaction with the entry receptor, ACE2, which may allow for stronger binding and infectivity [13,15]. 

A limitation to be considered in the context of this study was the choice to focus on the three most pathogenic *Betacoronavirus* species for comparison, while not including analysis of the less pathogenic human *Betacoronaviruses*. A larger screen of viral mutations could potentially provide a broader and more holistic understanding of *Betacoronavirus* evolution. However, the data selected for this analysis were chosen for clinical relevance. Another limitation of this study was that evaluations of the viral mutations were performed at the peptide and protein levels and excluded genomic analysis of changes occurring at the nucleobase level. This method was chosen to be able to assess the changes in amino acid chemical properties and the physical properties of the expressed viral proteins as a way of understanding how the receptor and spike protein interactions may have changed at the biochemical level. This study was conducted as a snapshot of *Betacoronavirus* species evolution. As COVID-19 has become endemic for much of the global population, SARS-CoV-2 continues to mutate and adapt. Continuous proteomic and genomic analyses of these new variants would further be able to identify the molecular differences as they develop between SARS-CoV-2 variants to map viral evolutionary trees and provide methods to distinguish viral rates of change and the rapid implications of molecular evolution to infectivity and severity of disease for SARS-CoV-2 variants as they emerge.

## Conclusions

Our results demonstrate that the SARS-CoV-2 pathogenic *Betacoronavirus* displayed the largest magnitude difference in charge between the viral spike protein receptor-binding domain versus its receptor, ACE2, suggesting the potential for a stronger electrostatic binding interaction. Surface area measurements of each viral RBD show that SARS-CoV-2 also had the largest RBD surface area compared to SARS-CoV and MERS-CoV, indicating more area for molecular interaction with its receptor, ACE2. Viral evolution of SARS-CoV-2 to possess a larger and more electrostatically “sticky” RBD compared to other pathogenic *Betacoronavirus* species may contribute to observations of SARS-CoV-2 having a stronger or more stable binding, leading to the observed increase in viral transmissibility and infectivity during the COVID-19 pandemic. Further investigation of conserved genomic regions between these viruses may facilitate the development of viable vaccines and treatments to combat the current pandemic as well as potential future pathogenic mutations. After conducting an analysis of 100 spike protein sequences of SARS-CoV-2, several highly conserved regions were identified within the RBD that could be sites of interest for future studies as vaccine targets. Of note, residue 396-452 was the longest identified conserved region at 56 base pairs in length that could be an important area for consideration of vaccine development.
